# A microcomputed tomographic analysis of the morphological variabilities and incidence of extra canals in mandibular first molar teeth in an Egyptian subpopulation

**DOI:** 10.1038/s41598-023-36005-7

**Published:** 2023-06-02

**Authors:** Shehabeldin Mohamed Saber, Mohamed Mohamed Elashiry, Shaimaa Mohamed Abu El Sadat, Nawar Naguib Nawar

**Affiliations:** 1 Department of Endodontics, Faculty of Dentistry, The British University in Egypt, 81-11-11 El-Rehab, Cairo, 11841 Egypt; 2 The Center for Innovative Dental Sciences, Faculty of Dentistry, The British University in Egypt, Cairo, Egypt; 3 Department of Endodontics, Faculty of Dentistry, Ain Shams University in Egypt, Cairo, Egypt; 4 Department of Endodontics, Dental College of Georgia, Augusta University, Cairo, Egypt; 5 Department of Oral Radiology, Faculty of Dentistry, Ain Shams University in Egypt, Cairo, Egypt

**Keywords:** Preclinical research, Dental pulp, Root canal treatment

## Abstract

A well-protected microbial habitat may be present in the root and canal morphology, which is varied and complicated. Before initiating effective root canal treatment, a detailed knowledge of the root and canal anatomical variances in each tooth is a must. This study aimed to investigate the root canal configuration, apical constriction anatomy, location of the apical foramen, dentine thickness, and prevalence of accessory canals in mandibular molar teeth in an Egyptian subpopulation using micro-computed tomography (microCT). A total of 96 mandibular first molars were scanned using microCT, and 3D reconstruction was performed using Mimics software. The root canal configurations of each of the mesial and distal root were classified with two different classification systems. The prevalence and dentin thickness around middle mesial and middle distal canals were investigated. The number, location and anatomy of major apical foramina and the apical constriction anatomy analysed. The number and location of accessory canals were identified. Our findings showed that two separate canals (15%) and one single canal (65%) were the most common configuration in the mesial and distal roots, respectively. More than half of the mesial roots had complex canal configurations and 51% had middle mesial canals. The single apical constriction anatomy was the most common for both canals followed by the parallel anatomy. Disto-lingual and distal locations of the apical foramen are the most common location for both roots. Mandibular molars in Egyptians show a wide range of variations in root canal anatomy with high prevalence of middle mesial canals. Clinicians should be aware of such anatomical variations for successful root canal treatment procedures. A specific access refinement protocol and appropriate shaping parameters should be designated for each case to fulfil the mechanical and biological objectives of root canal treatment without compromising the longevity of treated teeth.

## Introduction

The root and canal morphology are variable and complex and can harbor a well-protected microbial environment^1^. A thorough knowledge and meticulous understanding of the root and canal anatomical variations in every tooth type is a prerequisite before commencing successful root canal treatment procedures^1,2^. Root and canal morphology in different populations have been the subject of many experimental and clinical studies using a wide variety of techniques including clearing and staining method, 3D imaging techniques involving cone beam computed tomography (CBCT) and micro-computed tomography (microCT)^3–6^.

MicroCT technology is an accurate, nondestructive research tool that offers high resolution qualitative images and quantitative analysis of the root canal system^4^. It provides details on the root (danger zone) thickness^7^, root canal configurations^4^, intercanal communications^8^, accessory canals^9^ and anatomy of the root apex including apical foramen and apical delta^10,11^. Literature shows that the internal and external root anatomy of mandibular molars is highly variable^3,5,11,12^. The number of roots ranges from one to four, and many root canal configuration types have been reported^3,4,11^. Recent microCT studies also showed growing body of knowledge with regards to root thickness^7^, root canal isthmus^8^, prevalence of accessory canals^13^ and detailed anatomy of the root apex^11^.

A new classification system for root and canal anatomy has been proposed which provides detailed information on tooth notation, number of roots and root canal configuration in addition to accessory canals and dental anomalies^14–17^. This coding system overcomes deficiencies in previous systems by addressing the number of roots in every tooth type and has the ability to describe root canal configurations without referring to specific Roman numerals, which is a challenge when applying the Vertucci classification to teeth with complex canal systems^14,18^. Recent surveys amongst dental students and dental practitioners have supported the application of the new coding system in teaching, research, and clinical practice^19,20^.

To date, information on the root canal systems of mandibular molars in Egyptian population using microCT is scarce. This study aims to investigate the root canal configurations, prevalence of middle mesial and middle distal canals and the radicular dentin thickness in relation to them, number, location and anatomy of the major apical foramina, anatomy of the apical constriction in addition to number and location of accessory canals in extracted double rooted, mandibular first molars in an Egyptian subpopulation using microCT technology.

## Materials and methods

All methods were carried out in accordance with relevant guidelines and regulations. The protocol was approved by the research ethics committee of the faculty of Dentistry, Ain shams University (FDASU-RECID-021607).

### Preparation of the study samples

Mandibular first molars extracted for reasons not related to this study were selected from an Egyptian subpopulation. Gender and age were unknown. An informed consent was obtained from all subjects and/or their legal guardian(s) to use their extracted teeth for scientific purposes instead of being incinerated. Teeth with root caries, previous root canal treatment, immature apices or root resorption were excluded. A sample size calculation was performed using a sample size calculator (https://www.calculator.net/sample-size-calculator.htm). Egypt has a population of about 110 million individuals. 90% level of confidence and 9% margin of error resulted in a sample size of 85 mandibular first molar teeth. A total of 96 samples were included in this study.

All teeth were placed in 5.25% sodium hypochlorite for 30 min. Subsequently, the remaining soft tissues, bony fragments, and calculus (if present) were removed using an ultrasonic scaler (Neutron P5, Satelec, Acteon, North America). The samples were then stored in saline at room temperature until use.

### Micro-computed tomography

Teeth were fixed on mounting stubs, and microCT acquisitions with a resolution of 27 μm were obtained by using Inveon Multimodality Single photon emission computed tomography–CT scanner (Siemens Preclinical Solutions, Knoxville, TN). Reconstruction of the images was done using NRecon software (SkyScan 1174, SkyScan, Bruker, Belgium), the ring artifact correction was set at 10, and beam hardening correction was set at 15. The 3-dimensional image reconstruction was performed using Mimics Medical software version 21.0.0.406 (Materialise NV, Technologielaan 15, 3001 Leuven, Belgium). The data were inspected by three observers. Disagreement in the interpretation of images was discussed between a fourth observer until a consensus was reached^21,22^.

The following objectives were examined:

1. Root canal configuration types using two systems (Vertucci^3^, Ahmed et al.^14^), including the prevalence of middle mesial and middle distal canals (MMC & MDC).
2. Prevalence of middle mesial (MMC) and middle distal (MDC) canals, their classification according to Pomeranz et al.^23^, their largest and smallest mesiodistal diameter along their course, as well as the minimal radicular dentin thickness in relation to them because of its clinical significance.
3. Number, location, and anatomy of the major apical foramina.
4. Apical constriction anatomy (single, parallel, tapered, flaring and delta)—according to Divine et al.^24^.
5. Presence and location of accessory canals.

### Images calibration for measurements related to the MMC and MDC

To acquire accurate measurements of the canals’ diameters and the minimal radicular dentin thickness related to them, the method detailed by Saber et al. was adopted^25^. *On the axial view****:*** the axial plane was adjusted just below the furcation area, and each root was measured separately. Reference planes were adjusted so that sagittal plane bisects the root B-L and the coronal plane bisects the root M-D. *On the sagittal view:* The coronal plane was adjusted to pass through the apical 1/3 of the tooth and bisect the root M-D. *On the coronal view:* the sagittal plane was adjusted to bisect the root along the long axis passing by the root apex. Finally, measures were taken every 1 mm starting from the most cervical point where an MMC or MDC is evident moving apically.

### Statistical analysis

Categorical data were presented as frequency and percentage values and were analyzed using chi-square test followed by pairwise comparisons utilizing multiple z-test with Bonferroni correction. The significance level was set at 0.05 (p < 0.05). As for middle mesial and middle distal canals, categorical data were presented as frequency and percentage values, while numerical data were presented as mean, standard deviation (SD), median and interquartile range values. Statistical analysis was performed with R statistical analysis software version 4.1.2 for Windows. Intra-observer reliability was checked using the Wilcoxon signed-rank test, while inter-observer reliability was assessed using the Cohen kappa test. Significance was set at 0.05 (*P* < 0.05).

## Results

### Root canal configurations in the mesial and distal roots

**Figure 1:**
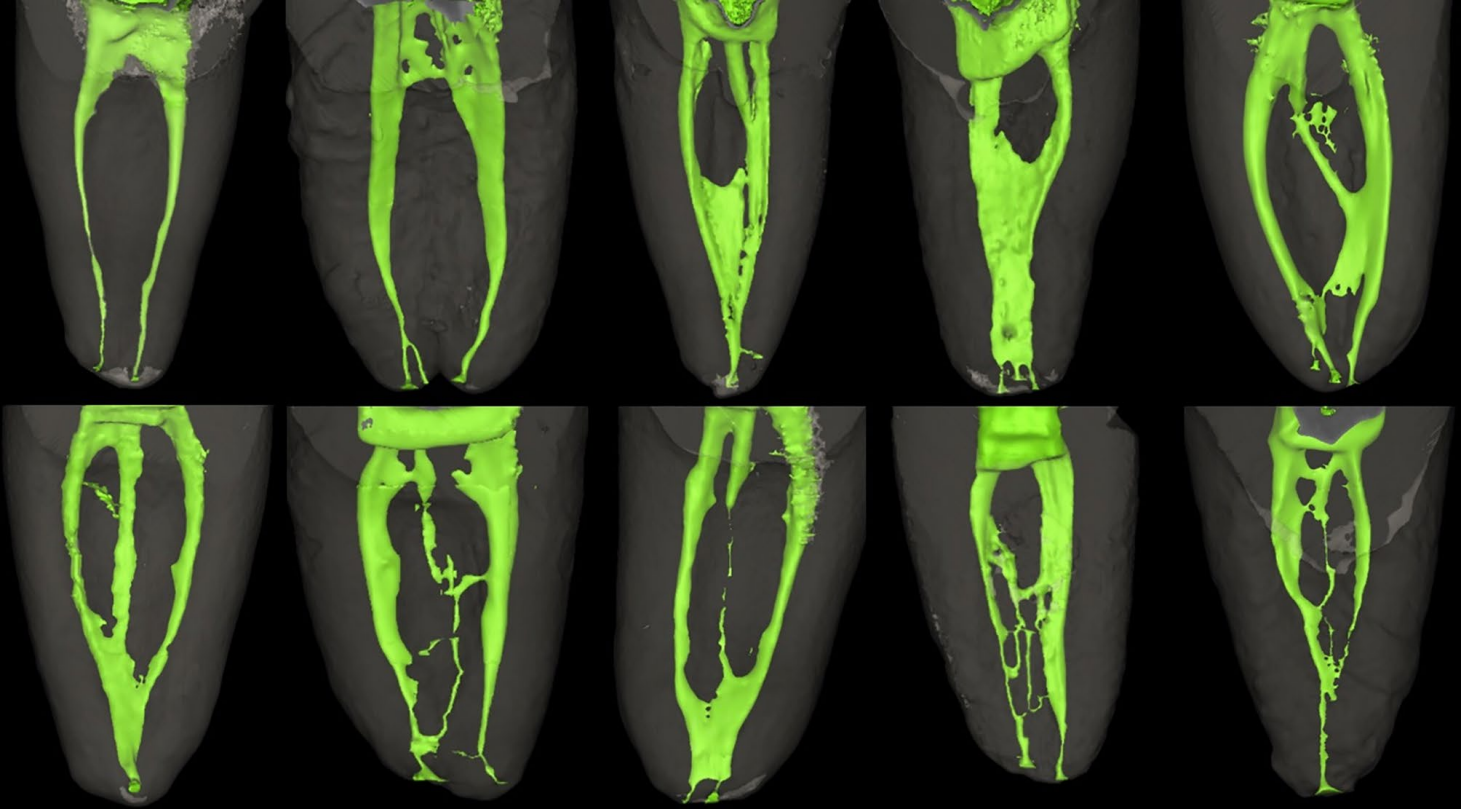
Micro-CT scanning showing different root canal configuration types in the mesial root of mandibular molar teeth.

Root canal systems were firstly identified according to Vertucci^3^ as well as Ahmed et al*.*^14^. The identified configurations were then classified according to the number of digits comprising their code into simple, more complex and severely complex. The mesial root showed a wide variation in its anatomy as the root canal systems were categorized into 39 possible root codes, only 5 of which were classifiable according to Vertucci^3^. Root canal morphology of the mesial root is described according to complexity in Tables 1, 2 and 3 and samples of the constructed images are shown in Fig. 1.

**Table 1:** Root code, number and percentage of root canal morphology in the mesial root of double rooted mandibular molars—simple configurations (2 digits).

2 digits simple configurations								
Root code	M^2^	M^2–1^	M^1–2^	M^1–2–1^	M^2–1–2^	M^3–1^	M^3–2^	M^2–3^
(Corresponding Vertucci’s Type)	(IV)	(II)	(V)	(III)	(VI)	(N/A)	(N/A)	(N/A)
Number & percentage	17 (17.71%)	8 (8.33%)	1 (1.04%)	7 (7.29%)	3 (3.13%)	3 (3.13%)	3 (3.13%)	3 (3.13%)
Total	45 (46.88%)							

(N/A); not available.

**Table 2:** Root code, umber and percentage of root canal morphology in the mesial root of double rooted mandibular molars—canals of more complex configurations (3 and 4 digits). All root canal configurations in this table are unclassifiable via Vertucci’s classification.

3 digits complex configurations										
Root code	M^1–2–3^	M^3–2–1^	M^2–3–2^	M^3–2–3^	M^2–3–1^	M^2–4–2^	M^2–4–3^	M^3–4–3^	M^4-3-2^	M^4-2-3^
Number & percentage	1 (1.04%)	5 (5.21%)	4 (4.17%)	1 (1.04%)	3 (3.13%)	1 (1.04%)	1 (1.04%)	1 (1.04%)	2 (2.08%)	1 (1.04%)
Total	20 (20.83%)									
4 digits complex configurations										
Root code	M^1–3–2–1^	M^2–3–2–1^	M^2–3–2–3^	M^3–2–3–2^	M^3–2–3–4^	M^3–2–1–2^		M^3–2–3–4^		M^3–4–3–2^
Number & percentage	3 (3.13%)	5 (5.21%)	2 (2.08%)	2 (2.08%)	1 (1.04%)	2 (2.08%)		1 (1.04%)		1 (1.04%)
Total	17 (17.71%)

**Table 3:** Root code, number and percentage of root canal morphology in the mesial root of double rooted mandibular molars—canals of severely complex configurations (5 digits and more). All root canal configurations in this table are unclassifiable via Vertucci’s classification.

5 digits complex configurations												
Root code	M^2–3–2–3–2^	M^1–2–3–2–1^		M^2–3–2–1–2^		M^3–4–5–3–1^		M^2–3–4–3–2^		M^2–3–2–1–3^		M^2–3–2–1–2^
Number & percentage	1 (1.04%)	2 (2.08%)		1 (1.04%)		1 (1.04%)		1 (1.04%)		1 (1.04%)		1 (1.04%)
Total	8 (8.33%)											
More than 5 digits complex configurations												
Root code	M^2–3–2–1–2–1^		M^2–3–2–1–3–2^		M^1–2–3–2–3–2^		M^3–1–2–1–2–1^		M^3–2–3–2–3–2–3–2^		M^2–3–4–2–1–2–3^	
Number & percentage	1 (1.04%)		1 (1.04%)		1 (1.04%)		1 (1.04%)		1 (1.04%)		1 (1.04%)	
Total	6 (6.25%)

**Figure 2:**
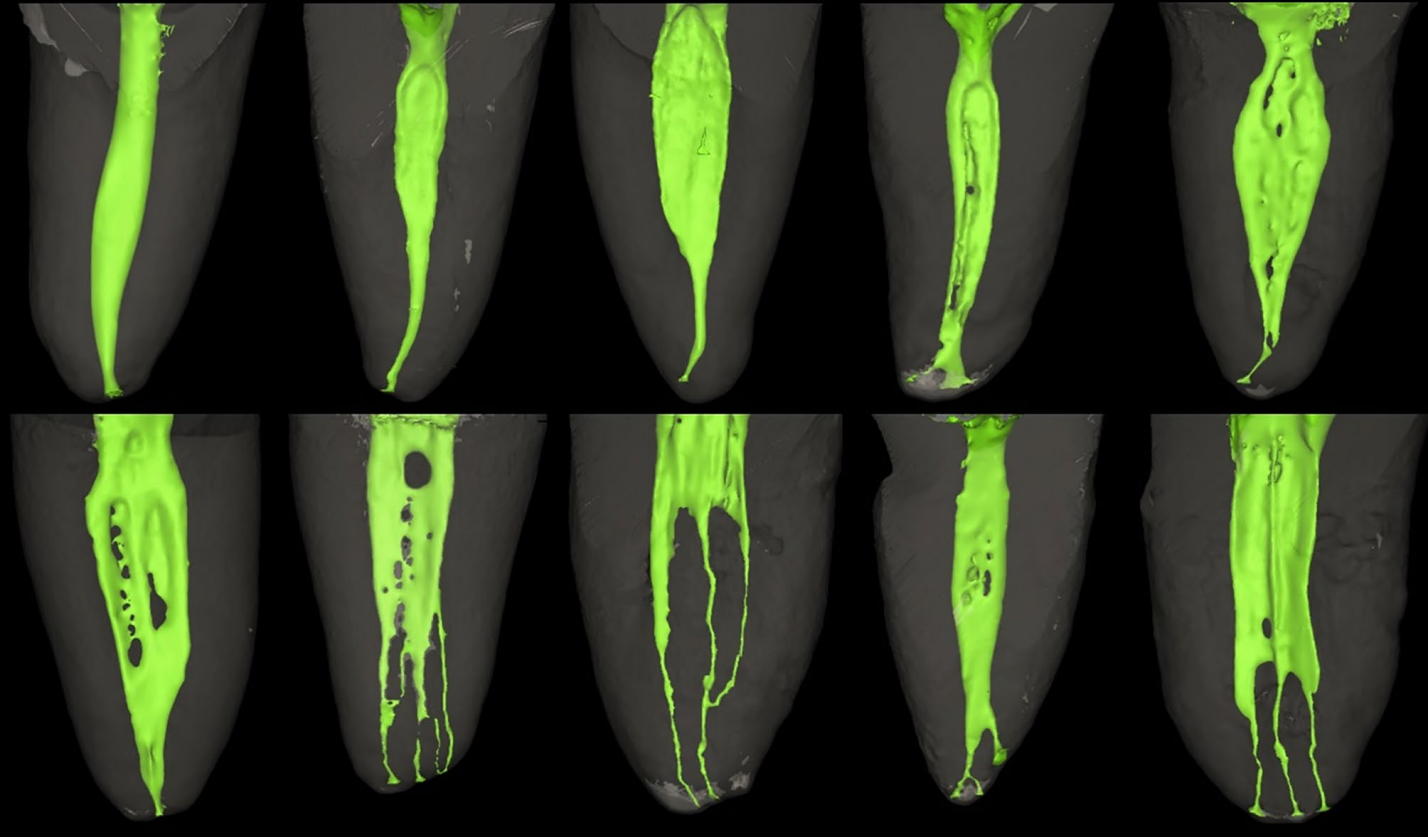
Micro-CT reconstructed images showing the root canal morphology of the distal root in mandibular molar teeth.

Root canal morphology of the distal root showed less variation than the mesial root with its root canal systems categorized into 20 possible root codes only four of which followed the Vertucci classification^3^. Root canal morphology of the distal root is described according to complexity in Tables 4 and 5 and samples of the constructed images are shown in Fig. 2.

**Table 4:** Root code, number and percentage root canal morphology in the distal root of double rooted mandibular molars—canals of simple and more complex configurations.

Simple configurations					
Root code	D^1^	D^1–2^	D^1–2–1^	D^1–2–1–2^	D^2–1–2–1^
(Corresponding Vertucci’s Type)	(I)	(V)	(III)	(VII)	(N/A)
Number & percentage	65 (67.71%)	3 (3.13%)	7 (7.29%)	2 (2.08%)	1 (1.04%)
Total	78 (81.25%)				
Complex configurations (unclassifiable via Vertucci’s classification)					
Root code	D^3–1^	D^3–2^	D^1–3–1^	D^3–1–2^	D^1–2–5^
Number & percentage	1 (1.04%)	1 (1.04%)	1 (1.04%)	1 (1.04%)	1 (1.04%)
Total	5 (5.21%)				

(N/A); not available.

**Table 5:** Root code, number and percentage root canal morphology in the distal root of double rooted mandibular molars—canals of severely complex configurations (4 digits and more). All root canal configurations in this table are unclassifiable via Vertucci’s classification.

4 & 5 digits complex configurations					
Root code	D^1–3–2–1^	D^1–2–3–1^	D^1–2–3–4–3^	D^2–3–2–1–3^	D^1–2–1–2–1^
Number & percentage	2 (2.08%)	2 (2.08%)	1 (1.04%)	1 (1.04%)	1 (1.04%)
Total	7 (7.28%)				
More than 5 digits complex configurations					
Root code	D^1–2–3–2–1^	D^1–2–3–4–3–2–1^	D^2–3–2–1–2–3^	D^2–3–2–3–2–1–2–1^	D^1–2–3–4–3–2–1–2–1^
Number & percentage	1 (1.04%)	2 (2.08%)	1 (1.04%)	1 (1.04%)	1 (1.04%)
Total	6 (6.25%)

### Middle mesial and middle distal canals and minimal radicular dentin thickness in relation to them

**Figure 3:**
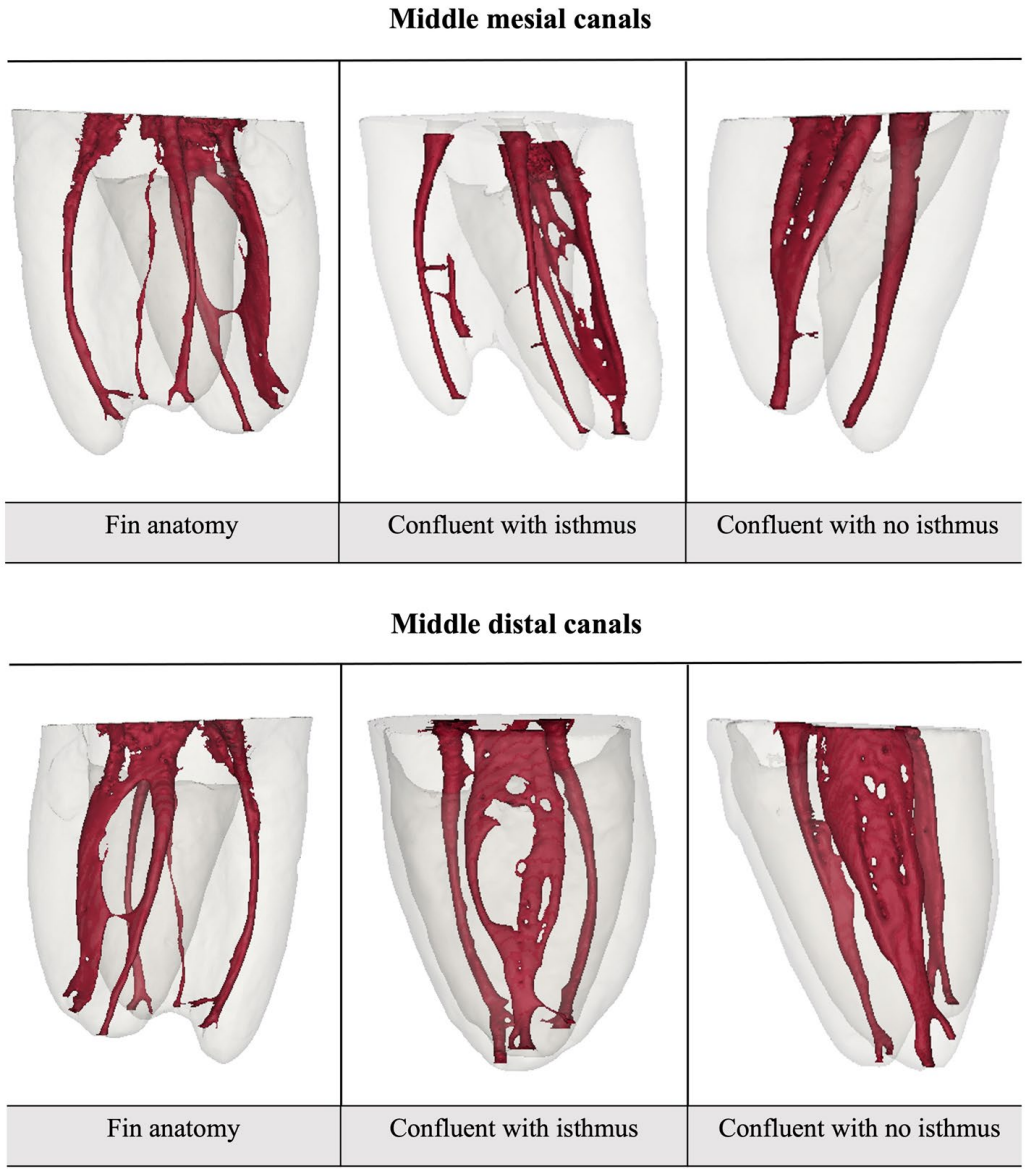
Representative samples of the different types of MMC and MDC observed (reconstruction was performed using Mimics Medical software version 21.0.0.406 (Materialise NV, Technologielaan 15, 3001 Leuven, Belgium).

Number and classification of MMC and MDC is shown in Table 6. For both canals the most commonly found anatomies were the “confluent with isthmus” and the “confluent without isthmus”, and the difference between them was insignificant. Also, the “independent” anatomy was not found for either canal. Parameters concerning MMC and MDC diameters and the radicular dentin thickness in relation to them are shown in Table 7. Representative samples of the MMC and MDC observed in this study are shown in Fig. 3.

**Table 6:** Canal configuration in middle mesial and middle distal canals.

Canal	Independent anatomy	Fin anatomy	Confluent with isthmus	Confluent without isthmus	Double	Total
Middle mesial						
n	0	7	20	17	5	49
%	0.0%	14.3%	40.8%	34.7%	10.2%	100%
Middle distal						
n	0	2	6	6	1	15
%	0.0%	13.3%	40.0%	40.0%	6.7%	100%

n; number.

**Table 7:** Descriptive statistics for different parameters in middle mesial and middle distal canals.

Canal	Parameter	Mean	95% CI		SD	Median	IQR
			Lower	Upper			
Middle mesial	Minimal mesial and distal dentin thickness relative to MM in mm						
	Minimum distance to mesial wall	1.05	0.95	1.16	0.40	0.98	0.46
	Minimum distance to distal wall	0.99	0.89	1.09	0.38	0.93	0.49
	Smallest and largest mesiodistal diameter						
	Smallest diameter	0.17	0.15	0.20	0.08	0.17	0.12
	Largest diameter	0.33	0.30	0.36	0.11	0.31	0.16
	Minimal mesial and distal dentin thickness relative to MD in mm						
	Minimum distance to mesial wall	1.21	1.05	1.36	0.33	1.06	0.56
	Minimum distance to distal wall	1.40	1.25	1.56	0.33	1.39	0.39
Middle distal	Smallest and largest mesiodistal diameter						
	Smallest diameter	0.26	0.22	0.31	0.09	0.25	0.11
	Largest diameter	0.37	0.33	0.42	0.10	0.35	0.15

MM; middle mesial, MD; middle distal, 95% CI; 95% confidence interval for the mean; SD; standard deviation, IQR; interquartile range.

### Apical foramina

#### Number and location

**Figure 4:**
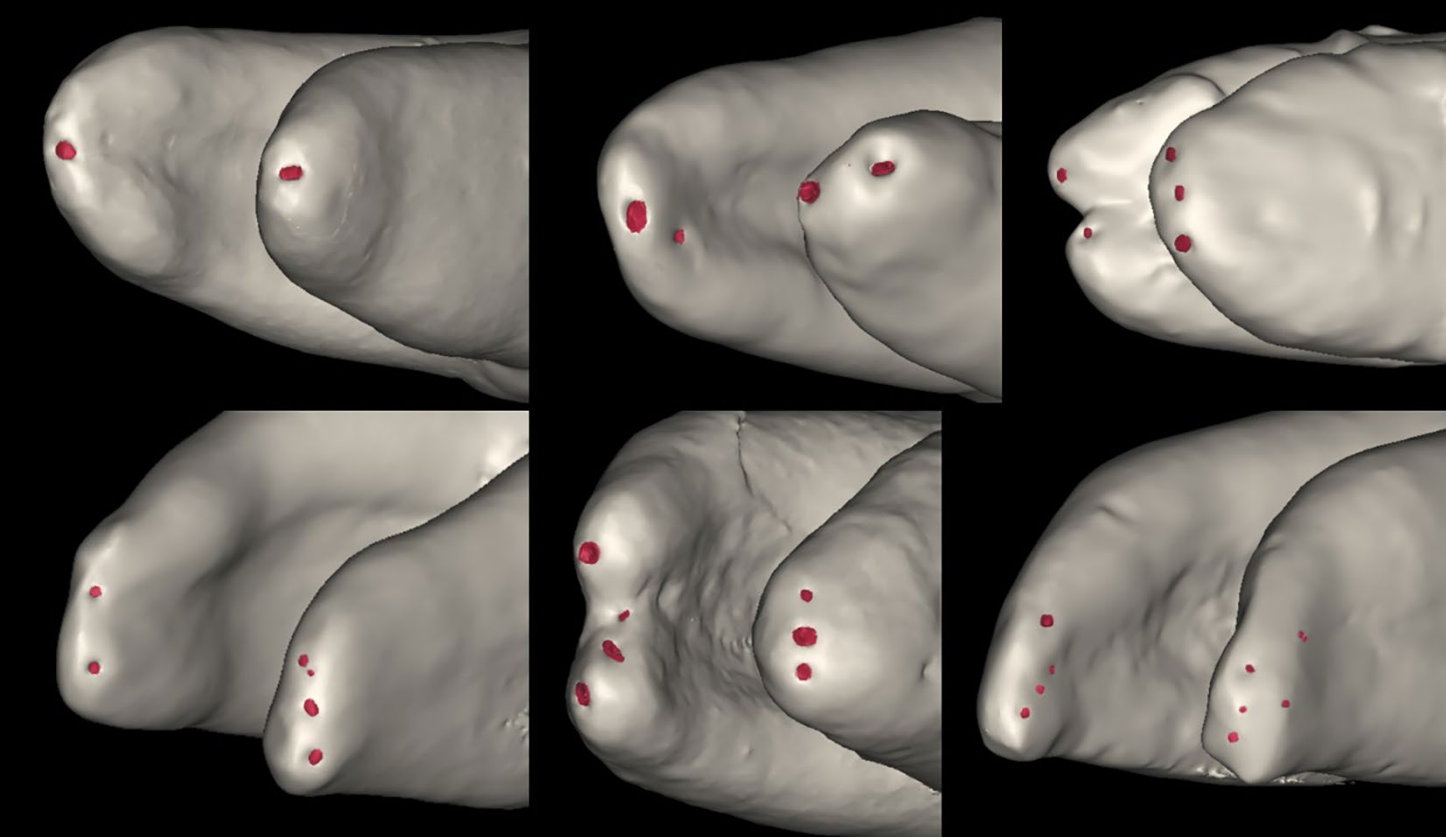
Micro-CT reconstructed images showing different locations of the apical foramina in the mesial and distal roots of mandibular molars.

Most of the mesial roots showed two apical foramina while the majority of distal roots showed a single foramen (Table 8, Fig. 4). The location of the apical foramen in relation to the radicular apex showed a wide variation (Table 9, Fig. 4).

**Table 8:** Number of major apical foramina in the mesial and distal roots. Data presented in number and percentages.

Number of major apical foramina	Mesial		Distal		p-value
	n	%	n	%	
One	27	27.8^A^	60	61.9^B^	< 0.001*
Two	37	38.1	22	22.7	
Three	18	18.6	10	10.3	
Four	8	8.2	4	4.1	
Five	6	6.2	0	0.0	
Six	0	0.0	1	1.0	
Seven	1	1.0	0	0.0	

Values with different superscript letters within the same horizontal row are significantly different *; significant (*p* < 0.001), n; number.

Significant values are in [bold].

**Table 9:** Location of the major apical foramina in the mesial and distal roots. Data presented in number and percentages.

Root	Location							
	M	D	B	L	MB	ML	DB	DL
Mesial root (ML)	11 (13.58%)	16 (19.76%)	2 (2.47%)	7 (8.64%)	2 (2.47%)	6 (7.41%)	18 (22.22%)	19 (23.45%)
Mesial root (MB)	10 (12.35%)	21 (25.92%)	6 (7.41%)	2 (2.47%)	14 (17.28%)	6 (7.41%)	12 (14.81%)	10 (12.35%)
Mesial root (Canal 2–1)	2 (5.27%)	9 (23.69%)	2 (5.27%)	3 (7.89%)	3 (7.89%)	3 (7.89%)	8 (21.05%)	8 (21.05%)
Distal root	13 (10.93%)	38 (31.94%)	5 (4.21%)	9 (7.56%)	8 (6.72%)	16 (13.44%)	16 (13.44&)	14 (11.76%)

M; mesial, D; distal, B; buccal, L; lingual, MB; mesiobuccal, ML; mesiolingual, DB; distobuccal, DL; distolingual.

1.64% of the Samples had a single mesial canal ended in a delta with foramina on the distal side. The sample was not included in the above statistics.

5.49% of the samples (5 distal roots) had two distinct canals that were considered separately for the above statistics. In such cases, 100% of DB canals foramina showed distally. On the other hand, the DL canal distribution showed a little bit more variation with 33.34% of the foramina showing distolingually.

#### Apical constriction anatomy

**Figure 5:**
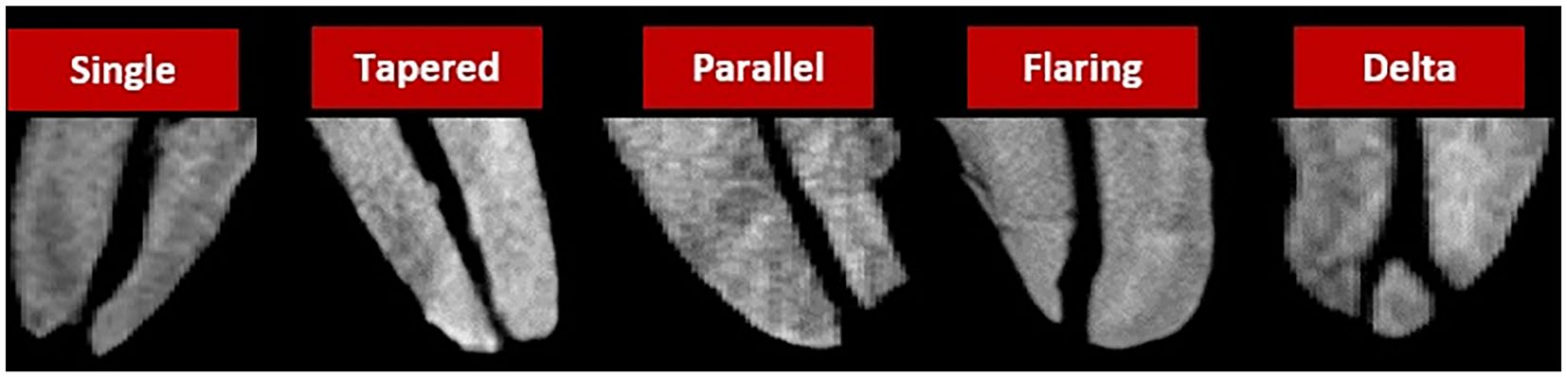
Micro-CT images showing different types of apical foramen morphology.

The anatomical classification for the apical foramina is shown in Table 10 and representative samples are shown in Fig. 5.

**Table 10:** Anatomy of the apical foramina in the mesial and distal roots. Data presented in number and percentages.

Root	Apical foramina configurations				
	Single	Tapering	Parallel	Flaring	Delta
Mesial root (ML)	22 (37.93%)	12 (20.60%)	9 (15.51%)	9 (15.51%)	10 (17.24%)
Mesial root (MB)	31 (53.45%)	6 (10.34%)	10 (17.24%)	5 (8.62%)	10 (17.24%)
Mesial root Canal (2–1)	12 (31.58%)	5 (13.58%)	4 (10.52%)	14 (36.84%)	3 (7.89%)
Distal root	44 (48.36%)	10 (10.98%)	12 (13.19%)	12 (13.19%)	13 (14.28%)

For the mesial root, 41% of the samples (58 mesial roots) have distinct MB and ML canals, while 39.5% (38 mesial root) have canal (2–1). For the distal root, 5.49% of the samples (5 distal roots) had two distinct canals that were considered separately from the below data. DB showed single, parallel and delta anatomies while DL canal showed single, flaring and delta anatomies. For both canals, the single anatomy was slightly more.

### Presence and location of lateral canals

For the mesial root, 27.83% of the whole samples (27 mesial root) showed the presence of lateral canals. 7.22% of the samples (7 mesial root) showed more than one lateral canal at different canal levels. Total number of accessory canals in the mesial roots was 36. For the distal root, 25.77% of the total samples (25 distal root) showed the presence of lateral canals. 6.18% of the total samples (6 distal root) showed two lateral canals at different canal levels. Total number of accessory canals in the distal roots was 28 (Table 11).

**Table 11:** Number and location of accessory canals in the mesial and distal roots. Data presented in numbers and percentages.

Location of lateral canals	Mesial		Distal		p-value
	n	%	n	%	
Coronal	3	8.3	2	7.1	**0.317**
Midroot	22	61.1	12	42.9	
Apical	11	30.6	14	50.0	

n; number.

Significant values are in [bold].

## Discussion

A sound knowledge of root anatomy and canal morphology is important for successful root canal treatment^1^. High resolution microCT imaging has been used for the study of root canal morphology in different population groups^4,8,26^. This study investigated the root and canal anatomy of mandibular first molars in an Egyptian population using a recent canal coding system^14^.

The results of this study confirmed the expected variability in the internal root canal anatomy in mandibular first molars, which can contribute to endodontic treatment failures^27,28^. With regards to the mesial root, only 46.88% showed simple root canal configurations (that can be classified using Vertucci classification) while the rest of the samples showed different degrees of complexity, which were classified using Ahmed et al. coding system (non-classifiable Vertucci types)^3,14^.

The occasion of MMCs in 51% of the study samples is an interesting finding, which is comparable to the 46% incidence reported in one clinical study on patients with mixed ethnicities^29^ and another study on an Indian population^30^. This prevalence is probably one of the highest documented in the literature compared to other studies^31–33^ which is attributed to difference in methodologies (such as CBCT) or populations. However, it is worth mentioning that results of this study are in contrast with one microCT study performed on Egyptian mandibular first molars^34^. This could be explained by differences in sample size, microCT scanning parameters and the classification used for interpretation. Another CBCT study reported 25.6% of MMCs in mandibular first molars of Egyptians^35^. Indeed, the high accuracy of microCT scanning allows more detection of fine MMCs compared to CBCT.

The presence of MMCs is a clinical concern during the chemico-mechanical instrumentation in terms of the remaining dentin thickness and its susceptibility to fracture^36^. The presence of MMCs is more of a concern when compared to the MDC given the differences in anatomic features between the mesial and distal roots of a mandibular first molar in terms of curvature and dentin thickness^31^. In this study, the minimum dentin thickness relative to the MMC along the root canal length was found to be (1.05 ± 0.4) towards the mesial wall & (0.99 ± 0.38) towards the distal wall and the canal’s smallest diameter was found to be (0.17 ± 0.08) and its largest diameter was found to be (0.33 ± 0.11). These dimensions probably prioritize choosing shaping instruments with as maximum centering ability and minimum taper as practically possible. With regards to the distal root, the majority of the samples (81.25%) showed simple root canal configuration (in which can be classified using Vertucci classification) with 67.71% of the cases showing a single canal. These results are in agreement with two microCT studies^12,37^ performed on Brazilian and an American population. However, there is a difference in the second most common root canal configuration where in this study it was D^1–2–1^ (Vertucci type III), while it was D^2–1^ (Vertucci type II) in both the aforementioned studies.

Results of this study showed the Ahmed et al.^14^ classification system is more accurate and practical in classifying all canal configurations compared to Vertucci classification, in which a high percentage of the root canals in the mesial root did not fit into Vertucci classification. This agrees with several microCT and CBCT studies on different tooth types and population groups^18,38–40^.

Few studies investigated the apical foramina of mandibular molars using micro-CT^8,41,42^. Results showed that one or two foramina were identified in 66% and 84% of the mesial and distal roots, respectively. The remaining percentage showed up to seven foramina. This agrees with Fan et al.^41^, and in partial accordance with Marroquin et al.^43^ who also studied a sample of molars of Egyptian population and reported double or single foramina in most of their samples. However, the percentages reported by Marroquin et al.^43^ were higher than the ones in this study, and the highest number of foramina they reported in both roots was four. This may be attributed to the difference in methodology as their study was performed using stereomicroscope. Results of this study, however, confronts with Asijavičienė et al.^42^ who found that 74% of mesial roots had a single foramen. This contrast may be attributed to ethnical differences as their study was done in Lithuania though they did not explicitly identify the population examined.

This study investigated the location and the morphology of the apical foramina as well. Results showed that the “Single” configuration (according to Divine et al.^24^) was the most common for all canals of both roots except for when the mesiobuccal and mesiolingual canals merged into one foramen, where the “Flared” configuration was slightly more common than the “Single”. However, the other configurations “Tapering, Multiple and Delta” were still found in considerable percentages. These observations agree with Citterio et al.^44^ who reported that the apical constriction is a structure that constantly appears to be complex and variable. The location of the apical foramina usually varies and deviates from the apex. This agrees with previous studies^8,42,44,45^. This wide variations in locations from the long axis emphasizes the importance of electronic foramen locators to overcome inherent limitations of 2D radiographic imaging^42^.

In this study, there was no significant difference between the mesial and the distal roots in terms of the incidence of lateral canals. This is in contrast with Wolf et al.^34^ and Xu et al.^9^ who found that mesial roots of mandibular molars have significantly higher incidence of accessory canals. In the mesial roots of our study, 61% of the lateral canals were found in the middle one third followed by the apical one third (30%). In the distal root, half of the lateral canals were found in the apical one third, followed by the middle one third (43%). The coronal one third was the least portion to show lateral canals in both roots. This study did not investigate different morphological landmarks of accessory canals (such as tortuosity)^46^, which has little clinical implications^47,48^.

In terms of study limitations, the anonymity of the samples meant that correlating the findings to gender and age was not possible. Thus, we could not compare prevalence between genders, and we could not account for the age-related changes in the dimensions of the root canal system which can affect the overall frequency of MMC and MDC canals. This can be investigated in future studies. Also, this investigation was limited to the double rooted mandibular first molars. Prevalence of mandibular first molars having a third distolingual root (radix entomolaris/paramolaris) were previously studied in Egyptian population and was reported to be 3.12%^49^. Mandibular molars with three roots frequently fracture during extractions thus adding to the difficulty of obtaining sufficient representative samples for examination^50–52^.

## Conclusion

Mandibular first molars in Egyptians show a wide range of variations in the main and minor root canal anatomy as well as a high prevalence of middle mesial canals. Clinicians should be aware of such anatomical variations for successful root canal treatment procedures.

## Data Availability

The datasets generated during and/or analyzed during the current study are available from the corresponding author on reasonable request.
